# Leveraging syntactic and semantic graph kernels to extract pharmacokinetic drug drug interactions from biomedical literature

**DOI:** 10.1186/s12918-016-0311-2

**Published:** 2016-08-26

**Authors:** Yaoyun Zhang, Heng-Yi Wu, Jun Xu, Jingqi Wang, Ergin Soysal, Lang Li, Hua Xu

**Affiliations:** 1 School of Biomedical Informatics, University of Texas Health Science Center at Houston, Houston, TX 77030 USA; 2 School of Medicine, Indiana University, Indianapolis, IN 46202 USA

## Abstract

**Background:**

Information about drug–drug interactions (DDIs) supported by scientific evidence is crucial for establishing computational knowledge bases for applications like pharmacovigilance. Since new reports of DDIs are rapidly accumulating in the scientific literature, text-mining techniques for automatic DDI extraction are critical. We propose a novel approach for automated pharmacokinetic (PK) DDI detection that incorporates syntactic and semantic information into graph kernels, to address the problem of sparseness associated with syntactic-structural approaches. First, we used a novel all-path graph kernel using shallow semantic representation of sentences. Next, we statistically integrated fine-granular semantic classes into the dependency and shallow semantic graphs.

**Results:**

When evaluated on the PK DDI corpus, our approach significantly outperformed the original all-path graph kernel that is based on dependency structure. Our system that combined dependency graph kernel with semantic classes achieved the best F-scores of 81.94 % for in vivo PK DDIs and 69.34 % for in vitro PK DDIs, respectively. Further, combining shallow semantic graph kernel with semantic classes achieved the highest precisions of 84.88 % for in vivo PK DDIs and 74.83 % for in vitro PK DDIs, respectively.

**Conclusions:**

We presented a graph kernel based approach to combine syntactic and semantic information for extracting pharmacokinetic DDIs from Biomedical Literature. Experimental results showed that our proposed approach could extract PK DDIs from literature effectively, which significantly enhanced the performance of the original all-path graph kernel based on dependency structure.

## Background

Drug–drug interaction (DDI) is a condition where one drug alters the effect of another drug in a clinically meaningful way [1]. It is well documented to be one of the major causes of adverse drug reaction (ADR) and is thus, a demonstrated threat to public health [2–4]. With increasing rates of polypharmacy [5], the incidence of DDIs is likely to increase as well. Hence, collecting information about DDIs in a timely manner is critical for reducing ADR and the costs associated with therapy [6, 7]. Although significant efforts have been invested to incorporate DDIs into various data sources, such as DiDB [8], DrugBank [9], and pharmacy clinical decision support systems [10], existing sources suffer from the problems of low coverage [11], low accuracy [12] and low agreement [13].

Under such circumstances, scientific evidence detailing the mechanism/s behind the drug interactions are necessary to provide support for reliable DDI information [14]. To this end, FDA requires in vivo and in vitro DDI studies during new drug development [15, 16]. Since new reports of DDIs are rapidly accumulating in the huge archive of scientific literature [17], text mining techniques are needed to automatically extract DDIs with support from literature-derived scientific evidence [11].

A major type of DDI, PK DDI, is a situation wherein one drug affects (inhibits or induces) the absorption, distribution, metabolism, and/or excretion of another drug. Although mechanistic information regarding PK DDI provides important evidence by describing how the interaction between drugs occurs, very few studies have been conducted so far to extract PK DDIs from scientific literature. Currently, most DDI systems are built on the corpus that was used in the two DDI extraction challenges in 2011 and 2013 [18, 19]. However, a large part of this corpus is based on DrugBank. Only 86 DDI relations of PK mechanisms were annotated from Medline. In addition, [20] attempted to identify PK DDIs from drug package inserts. The texts taken from DrugBank and drug package inserts were manually curated with short and concise sentences, thus providing a brief description of DDIs [21]. In contrast, the scientific language used in literature typically contains long and complex sentences, expressing detailed PK information. Moreover, the content of scientific literature does not necessarily talk about DDIs, making DDI extraction from scientific literature significantly more difficult [21]. Other groups extracted the relation between drugs and enzymes based on properties of drug metabolism; here, potential DDIs were detected by inference and reasoning [22, 23]. The only DDI corpus dedicated to PK evidence derived from literature was built by Wu, Karnik et al. [24], covering both in vivo and *in vitri* PK DDI studies.

Promoted by the two DDI extraction challenges in 2011 and 2013 [18, 19], many approaches have been proposed to extract DDIs from biomedical text. The DDI extraction tasks are usually modeled as a classification problem. Machine learning (ML) methods were employed to classify whether the relation between each candidate DDI pair was a true interaction or not. In the existing ML-based systems, two types of methods have been mainly used: feature-based methods and kernel-based methods [25].

In feature-based methods, each data instance is represented as a feature vector in n-dimensional space. Features are defined to informatively represent the data characteristics of different relation types. Heterogeneous features of different linguistic levels have been employed in DDI extraction systems, including lexical features like negative words, syntactic patterns, semantic types of two drugs and ontology-based concepts [26–30]. In kernel-based methods, data instances are first represented by syntactic structures, using either the syntactic parse tree [31] or the dependency graph [32]. The similarity between the syntactic structural representations is then computed, as a representative of the similarity between the two instances. Various syntactic representations, similarity functions, and combinations are exploited in existing kernel-based DDI extraction systems [24, 26, 33, 34]. Bui Q-C et al. [25] leveraged both the syntactic structures and features of sentences, by using different feature lists according to different syntactic structures and achieved the best results on the challenge datasets. Currently, kernel-based methods are dominant and achieved state-of-the-art results for DDI [18, 19]. However, since scientific literature has many long and complex sentences, such approaches are likely to suffer from the sparseness problem of deep syntactic structures [35].

Also, sophisticated semantic information is rarely explored and employed for DDI. Semantic representations bearing more “compact” and generalized information could potentially normalize the surface form variations of syntactic structures. One important type of semantic information is predicate-argument-structures (PASs) [36]. PAS is a unified form of shallow semantic representation of the sentence, which is generated on the basis of variant syntactic structures [37]. PASs have already been used in various information extraction tasks and have shown promising results [38–40]. Another important type of semantic information is semantic class [41]. Based on the sublanguage theory [42, 43], semantic class is defined as the generic class of essential semantic information in the language of closed domains such as PK DDI, which is independent of the surface syntactic structures [41]. Sematic classes are different from the relatively high level semantic types defined in UMLS [44], which are currently used for DDI extraction. They are more granular, describing semantic information specific to a closed domain. For example, the word “strongly” in the sentence “Drug1 strongly increases plasma concentrations of oral drug2.” is an instance of the “Degree” semantic class and serves as a potential indication of the degree of PK DDI. However, it is not covered by UMLS as a concept. Many existing systems in different biomedical sub-domains used semantic class for relation extraction via rule-based semantic patterns [45, 46]. Nevertheless, semantic class hasn’t yet been examined for PK DDI extraction using statistical methods.

In this article, we examined the following two types of semantic information for PK DDI extraction from the biomedical text: shallow semantic representation and fine-granular semantic classes based on the sublanguage of PK DDI. All-path graph kernel was employed to statistically integrate different linguistic levels of information, syntactic, shallow semantic and fine-granular semantic class. Our approach differs from existing approaches in two ways. First, we propose a novel all-path graph kernel algorithm using shallow semantic graph, i.e. PAS graph kernel. Second, we statistically incorporate fine-grained semantic classes into dependency graph kernel and PAS graph kernel. Our evaluation results using the PK DDI corpus [24] demonstrates that our proposed approach significantly improves the performance of the original all-path graph kernel based on dependency structure.

## Results

### Performance of in vivo PK DDI extraction

Experimental results of PK DDI extraction for the in vivo dataset are displayed in Table 1. The PAS graph kernel outperformed the baseline dependency graph kernel with both higher precision (79.80 % vs. 78.79 %) and recall (76.06 % vs. 73.24 %). Further, when combined with semantic classes, the performance of dependency graph kernel increased significantly. The optimal F_1_ of 81.94 % was achieved by the dependency graph kernel with refined “mechanism” semantic classes. Semantic classes also enhanced the performance of PAS graph kernel (F_1_ 80.10 %). The refined semantic classes increased the precision of PAS graph kernel to 84.88 %. However, the recall dropped sharply to 68.54 %. Overall, the PAS graph kernel with refined semantic classes yielded the lowest F_1_ of 75.84 %.

**Table 1:** Performance for PK DDI extraction on the in vivo dataset

Methods	*P*	*R*	*F*_*1*_
DEP	78.79 %	73.24 %	75.91 %
PAS^a^	79.80 %	76.06 %	77.88 %
DEP_SC^a^	83.01 %	80.28 %	81.62 %
PAS_SC^a,b^	82.91 %	77.46 %	80.10 %
DEP_ReSC^a^	80.82 %	**83.10%**	**81.94 %**
PAS_ReSC^b^	**84.88 %**	68.54 %	75.84 %

Totally, six different methods were implemented. The abbreviation DEP stands for the dependency-based graph kernel, PAS stands for the graph kernel based on predicate-argument-structure, SC stands for semantic class information, and ReSC stands for refined semantic class information. DEP_SC means that semantic class information is incorporated into the dependency-based graph kernel. Precision (*P*), Recall (*R*) and F-measure (*F*_*1*_) were reported for each method. The highest performance under each evaluation criterion is bolded.

^a^ means the performance difference between the underlying method and DEP is statistically significant

^b^ means the performance difference between the underlying method and PAS is statistically significant. (p-value < 0.05)

### Performance of in vitro PK DDI extraction

Table 2 illustrates the experimental results of PK DDI extraction of the in vitro dataset. The baseline performance of dependency graph kernel was poor; the F_1_ was only 51.50 %. In contrast, PAS graph kernel got a 67.68 % F_1_. As observed for the in vivo dataset, the performance of the dependency graph kernel increased significantly with the incorporation of semantic classes. With refined semantic classes, the dependency graph kernel achieved the optimal F_1_ of 69.34 %. Semantic classes also consistently increased the performance of PAS graph kernel. Specifically, with refined semantic classes, PAS graph kernel obtained the highest precision of 74.83 %.

**Table 2:** Performance for PK DDI extraction on the in vitro dataset

Methods	*P*	*R*	*F*_*1*_
DEP	43.43 %	63.24 %	51.50 %
PAS^a^	73.03 %	62.07 %	67.68 %
DEP_SC^a^	70.32 %	61.93 %	65.86 %
PAS_SC^a,b^	69.23 %	66.48 %	67.83 %
DEP_ReSC^a^	70.76 %	**67.98%**	**69.34 %**
PAS_ReSC^a,b^	**74.83 %**	62.50 %	68.11 %

Totally, six different methods were implemented. The abbreviation DEP stands for the dependency-based graph kernel, PAS stands for the graph kernel based on predicate-argument-structure, SC stands for semantic class information, and ReSC stands for refined semantic class information. DEP_SC means that semantic class information is incorporated into the dependency-based graph kernel. Precision (*P*), Recall (*R*) and F-measure (*F*_*1*_) were reported for each method. The highest performance under each evaluation criterion is bolded.

^a^ means the performance difference between the underlying method and DEP is statistically significant

^b^ means the performance difference between the underlying method and PAS is statistically significant. (p-value < 0.05)

**Fig. 1:**
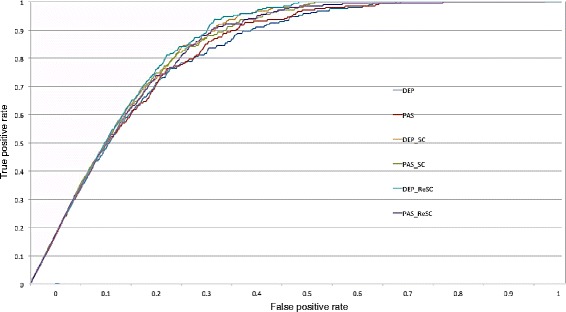
ROC curves of implemented methods on the in vivo dataset. The abbreviation DEP stands for the dependency-based graph kernel, PAS stands for the graph kernel based on predicate-argument-structure, SC stands for semantic class information, and ReSC stands for refined semantic class information. DEP_SC means that semantic class information is incorporated into the dependency-based graph kernel

**Fig. 2:**
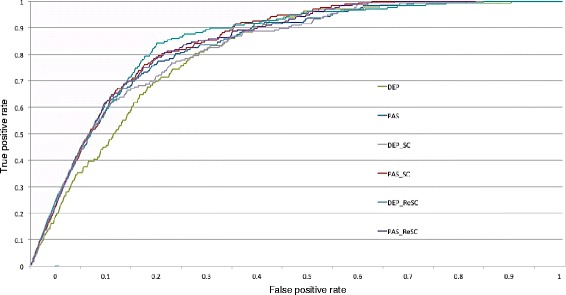
ROC curves of implemented methods on the in vitro dataset. The abbreviation DEP stands for the dependency-based graph kernel, PAS stands for the graph kernel based on predicate-argument-structure, SC stands for semantic class information, and ReSC stands for refined semantic class information. DEP_SC means that semantic class information is incorporated into the dependency-based graph kernel

Given that in real-world case the portion of negative DDI pairs is far higher than that of the positive ones, the ROC curves of implemented methods were also examined to check their sensitivity and specificity. Figures 1 and 2 illustrate the ROC curves on the in vivo and in vitro datasets, respectively. As can be seen from these figures, DEP_ReSC outperformed the other methods in terms of both sensitivity and specificity. Especially, a sharp enhancement over DEP by the other methods was observed from Fig. 2.

## Discussion

In this study, we examined the contribution of two types of semantic information for PK DDI extraction from literature. The shallow semantic representation, i.e., PAS of one sentence was employed as a novel alternative to dependency based syntactic structural representation in all-path graph kernel. Moreover, fine-granular semantic classes specifically designed as the sub-language for the closed domain of PK DDI were incorporated into dependency graph kernel and PAS graph kernel. Our results showed that both the types of semantic information improved the PK DDI extraction performance. PAS graph kernel outperformed the baseline of dependency graph kernel (in vivo: 77.88 % vs. 75.91 %; in vitro: 67.68 % vs. 51.50 %). Furthermore, integrating semantic classes into graph kernels achieved the optimal performance: dependency graph kernel got the optimal F_1_ (in vivo 81.94 %; in vitro 69.34 %), and PAS graph kernel yielded the highest precision (in vivo 84.88 %; in vitro 74.83 %). To the best of our knowledge, this is the first study that combines syntactic, shallow semantic and semantic class information into the graph kernel for PK DDI relation extraction.

### Performance variations between in vivo and in vitro datasets

**Fig. 3:**
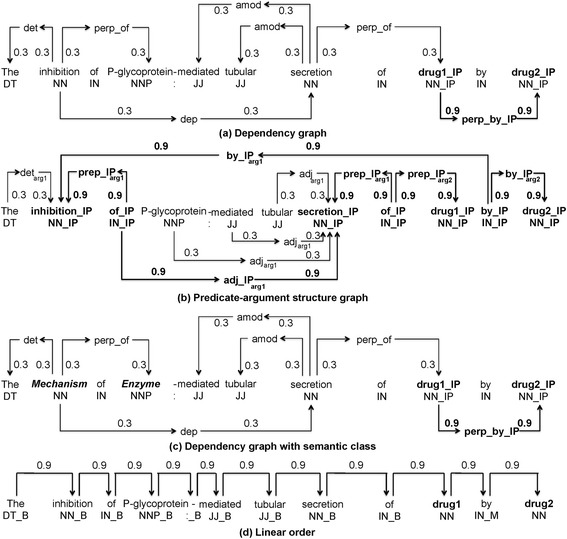
Illustration of multi linguistic level graph representation. The candidate interaction pair is marked as “drug1” and “drug2”. The shortest path between the drugs is shown in bold. In the dependency (a), predicate-argument structure (b), and an integration of semantic class with dependency (d) based subgraphs all nodes in the shortest path are specialized using a post-tag (IP). In the linear order subgraph (d) possible tags are (B)efore, (M)iddle, and (A)fter

As illustrated in Tables 1 and 2, the PK DDI performance on the in vivo and in vitro datasets have a significant difference. One of the major reasons is that literature about in vitro PK DDI contains more complex sentences with multiple clauses and conjunctive structures of drugs, making it more difficult to recognize DDI relations on the in vitro dataset. Nevertheless, as illustrated in Fig. 3, PAS captured more representative syntactic structural information of DDIs than DEP by considering the shallow semantic relations between syntactic constituents, especially when such syntactic constituents has a long-distance with the pair of drugs. Thus, it increased the precision on the in vitro dataset from 43.43 % to 73.03 %, in comparison to a precision enhancement from 78.79 % to 79.80 % on the in vivo dataset.

Another possible reason is the essential difference in literature description of the DDI evidence between in vivo PK DDI and in vitro PK DDI. In vivo PK DDI usually occurs when the exposure and efficacy of a probe drug is changed by another drug with the comparison of its pharmacokinetic parameters, while in vitro PK DDI occurs with the involvement of enzymes in the drug metabolism mechanism. For example, a DDI may occur when the metabolism of a probe drug is influenced by another drug, which is the inhibitor or inducer of the metabolizing enzyme. The distribution of the two types of evidence is different in these two corpora. This could explain an interesting observation that the precision of the in vivo dataset increased consistently by incorporating semantic classes from coarse to refined granularity with PAS (in vivo: PAS 79.80 %, PAS_SC 82.91 %, PAS_ReSC 84.88 %). In contrast, for the in vitro dataset, PAS_SC dropped the precision from PAS, while PAS_ReSC further increased the precision from PAS. (in vitro: PAS 73.03 %, PAS_SC 69.23 %, PAS_ReSC 74.83 %). The interaction mechanisms between drugs and enzymes present in the in vitro dataset are more diverse than in the in vivo dataset (Table 3). With only one single semantic class for mechanism, PAS_SC increased the recall while introducing more false positive predictions. On the contrary, PAS_ReSC enhanced the precision by differentiating among those mechanisms. That also explains why refining the semantic classes of mechanism yielded a relatively larger performance enhancement on the in vitro dataset (69.34 % vs. 65.83 %) than on the in vivo dataset (81.94 % vs. 81.62 %).

**Table 3:** Description of refined mechanism semantic classes for literature on PK DDI

Semantic class	Definition	Example
Drug-enzyme	The action of a drug on an enzyme	Inhibition
Enzyme-drug	The action of an enzyme on a drug	Catalyzes
Drug-metabolite	The action converting a drug to its metabolite	Hydroxylation

### Performance variations of different methods

As illustrated in Table 1 and 2, the PAS graph kernel achieved higher performance than the dependency graph kernel. Specifically in the in vitro dataset, the precision increased from 43.43 % to 73.03 % and F_1_ increased from 51.50 % to 67.68 %. This validated our assumption that more information to distinguish “DDI” from “NDDI” is covered by the paths of PAS graph kernel. With semantic class integration, the performance of both graph kernels increased. Nevertheless, the performance of dependency graph kernel was increased more sharply than PAS (in vivo: dependency 75.91 % vs. 81.62 %, PAS 77.88 % vs. 80.10 %; in vitro: dependency 51.50 % vs. 65.86 %, PAS 67.68 % vs. 67.83 %). On one hand, semantic information demonstrated its generalization ability to resolve the sparcity problem in syntactic paths. On the other hand, it also indicated that there was a relatively small gap between shallow semantic and semantic class representations of sentences.

As illustrated in Table 1, for the in vivo dataset, the improvement of DEP_ReSC was statistically significant over DEP. The performance of PAS_ReSC was comparable with DEP without statistically significant difference; whereas it dropped significantly from PAS. Moreover, as illustrated in Table 2, for the in vitro dataset, the improvement of DEP_ReSC was statistically significant over DEP; the improvement of PAS_ReSC was also statistically significant over DEP and PAS, respectively. Thus, refining semantic classes of “Mechanism” further enhanced the performance of dependency graph kernel. In contrast, the precision of PAS graph kernel was enhanced significantly by the refined semantic classes (in vivo: 82.91 % vs. 84.88 %; in vitro: 69.23 % vs. 74.83 %), with a severe drop in recall (in vivo: 77.46 % vs. 68.54 %; in vitro: 66.48 % vs. 62.50 %). One possible reason is that PAS graph kernel with refined semantic classes imposed strict constraints to patterns of positive DDIs, resulting in significantly increased precision at the cost of decreased recall.

### Error analysis

Table 4 lists the major reasons for false positive PK DDI recognition and the corresponding examples. Although negation expressions were already collected into the “Negation” semantic class and used to label the sentences, it still caused false positive errors, especially in sentences with complex structures (8 %). Another major reason observed in the in vitro PK DDI was that the relation between drug and its metabolites was misclassified as a DDI, because some trigger words of its metabolism mechanism are not covered in the training dataset (9 %). Some sentences described DDIs between drug pairs with cues of uncertainty, such as the word “whether” in the example sentence (7 %). Another reason for false positives was uncaught signs of comparison, such as the word “than” in the example (3 %). Besides, for long sentences with multiple clauses, the relations between irrelevant drug pairs across multiple clauses are prone to be misclassified (14 %).

**Table 4:** False positive error analysis of PK DDI extraction

Error categories	Example
Negation	Preincubation of human liver microsomes with **dihydralazine** in the presence of **NADPH** resulted in decreases in phenacetin O-deethylase activity (an indicator of P450 1A2 activity) and testosterone 6beta-hydroxylase activity (P450 3A4), but **not** in **diclofenac** 4′-hydroxylase activity (P450 2C9), an indication of inactivation of P450s 1A2 and 3A4 during the dihydralazine metabolism.
Relation between drug and its metabolites	In HLMs, **cisapride** was **N-dealkylated** to **norcisapride** (NORCIS) and hydroxylated to 3-fluoro-4-hydroxycisapride (3-F-4-OHCIS) and to 4-fluoro-2-hydroxycisapride (4-F-2-OHCIS).
Uncertainty	Because HMR1766 is an inhibitor and warfarin a substrate of CYP2C9, the authors studied **whether warfarin** pharmacokinetics and pharmacodynamics are influenced by **HMR1766**.
Comparison	The inductive effect of **CBZ** was about 46 % higher **than** that of **OXCZ**, a difference that may be of clinical relevance.
Cross-clause in long sentences	Coadministration with **ketoconazole** (which inhibits CYP3A4) decreased the mean apparent oral clearance of quinine significantly (P < .001) by 31 %, **whereas** coadministration with **fluvoxamine** (which inhibits CYP1A2 and to some extent CYP2C19) had no significant effect (P > .05) on the mean apparent oral clearance of quinine.

The drug names and important cue words in each example are bolded

Table 5 displays major reasons for false negative DDI recognition and the corresponding examples. As mentioned before, one crucial deficiency of dependency graph was that it failed to include critical DDI information into the shortest path, when two drugs were connected by prepositional structures. Some false negatives are caused by this deficiency (15 %). Another type of error was caused by conjunctive structures: usually only the relation between the first drug in the conjunctive structure and another drug was recognized, DDIs for the rest of the drugs were missed (14 %). Co-reference resolution was another cause of false negatives (6 %). In some cases, a numerical value change of PK parameters needed to be calculated first to determine the relation (5 %). Besides, literature may contain very rare relation patterns of DDIs, which were not covered in our current statistical model (13 %).

**Table 5:** False negative error analysis of PK DDI extraction

Error categories	Example
Relations failed to be covered by the shortest path of the graph	… suggesting that the degree of induction of **methadone metabolism by nevirapine** is similar for both dosing regimens…
Conjunctive structure	**Zafirlukast** inhibited the hydroxylation of **tolbutamide** (CYP2C9; mean IC(50) = 7.0 microM), **triazolam** (CYP3A; IC(50) = 20.9 microM) and **S-mephenytoin** (CYP2C19; IC(50) = 32.7 microM).
Co-reference resolution	Although **erythromycin** only modestly decreases lignocaine clearance, **it** causes a concomitant elevation of the concentrations of its pharmacologically active metabolite **MEGX**.
Need numerical calculation	Mean CYP2D6 **dextromethorphan** metabolic ratios before and after **fluoxetine** therapy were **0.028 +/-0.031 and 0.080 +/- 0.058,** respectively (P = .001)…
Rare relation pattern	The estimated K(i) values for CYP2D6-catalyzing **dextrorphan** formation were ranked in the following order: **perphenazine** (0.8 microM), **thioridazine** (1.4 microM), **chlorpromazine** (6.4 microM), **haloperidol** (7.2 microM), **fluphenazine** (9.4 microM), **risperidone** (21.9 microM), **clozapine** (39.0 microM), and **cis-thiothixene** (65.0 microM).

The drug names and important cue words in each example are bolded

### Limitations and future work

A limitation of this work is that currently the employed semantic classes are designed for PK DDIs, which may not be fully generalizable to other types of DDIs. Another limitation is that the current work focuses on recognizing interaction between two drugs. DDI may also be related to other important factors. For example, the existence of the protein NADPH is related to the interaction between dihydralazine and phenacetin in the sentence “Preincubation of human liver microsomes with dihydralazine in the presence of NADPH resulted in decreases in phenacetin O-deethylase activity”. Another important factor is the interaction between the drug and the enzyme, from which DDI relations not expressed explicitly in literature could be inferred. Moreover, drug targets interactions is also an important factor to consider for DDI extraction. If both of two drugs have interactions with the same target, they may have potential synergistic, additive or antagonistic interactions. Such factors would be considered for DDI relation extraction in our next step.

To further improve the performance of DDI relation extraction, a more accurate recognition of the negation expressions needs to be conducted. Whether those negations are modifying the DDI relations also need to be determined. To collect more trigger words for drug enzyme interaction and uncertainty, comprehensive semantic lexicons need to be built by leveraging existing knowledge resources such as UMLS and wordNet. Besides, specific strategy to handle different types of syntactic structures such as cross clauses relations, prepositional/conjunctive structures, and co-reference should be designed. One possible solution may be a hybrid way to combine statistical graph-kernel based methods with heuristic rules-based features, so that to consider simultaneouly the generalizability and specificity of the method.

What’s more, in the original annotation of the PK DDI corpus, DDIs can be further split into two types: certain DDIs with strong evidence and ambiguous DDIs with weak evidence [24]. Refinement of PK DDI relations according to different degrees of evidence will be carried out in our future work, to further leverage information from evidence for DDI recognition.

## Conclusions

In this study, two types of semantic information, shallow semantic representation and fine-grained semantic classes, were exploited for PK DDI extraction from biomedical text. All-path graph kernel was employed to statistically integrate different linguistic levels of information, i.e., syntactic, shallow semantic and fine-granular semantic class. Experimental results showed that our proposed approach significantly en-hanced the performance of the original all-path graph kernel based on dependency structure. The F-measure was improved from 75.91 % to 81.94 % on the in vivo dataset and from 51.50 % to 69.34 % on the in vitro dataset, respectively, demonstrating the potential of semantic information for effective PK DDI extraction.

## Methods

Two PK DDI datasets, consisting of in vivo and in vitro studies respectively, were used in this study. Our method consists of three steps. First, we represent sentences with syntactic structures, shallow semantic relation structures and semantic classes and their combinations. Second, all-path graph kernels describing the syntactic and semantic connections within the sentences are generated from those representations. In the last step, an SVM classifier is trained based on the graph kernels to generate a predictive model, which is used to classify candidate DDI pairs of the test dataset.

### Datasets

The corpus of PK DDI relations built by Wu, Karnik et al. [24] was employed in this study. The PK DDI relations was manually curated using 428 PK-DDI related abstracts from MedLine [24]. When searching for DDI studies from MedLine, the query “drug-drug interactions” was used by the DDI challenge corpus developers. In contrast, the PK DDI corpus of Wu, Karnik et al. [24] used additional keywords of probe substrate/inhibitor/inducers for specific metabolic enzymes in queries. The abstracts for annotation were randomly selected from the search results. In comparison with the PK DDIs (i.e., the “mechanism” relation) in the Challenge corpus, the PK DDI corpus is more focused on the co-occurrence of supportive evidence with a true positive DDI relation, such as drug enzyme mechanisms and changes in PK parameters. Furthermore, the abstracts in this corpus were categorized into two datasets for in vivo and in vitro studies, respectively, to accommodate the differences between the two study types. The datasets are described in detail below:

In vivo PK DDI dataset: 218 abstracts describing in vivo PK DDI studies are included in the dataset. In vivo PK DDI studies generally aim to determine the mechanism of potential interaction investigated, pharmacokinetics characteristics of drugs, mode of administration, and etc. To evaluate the effect of investigational drug on other drugs in in vivo studies, they typically apply crossover or sequential design experiments to investigate whether the exposure and efficacy of a probe drug is changed by another drug by comparing its pharmacokinetic parameters. Usually such parameters include Cmax, Tmax, and AUC, CL and the terminal half-life. An example sentence of in vivo PK DDI is shown in Table 6, in which the plasma concentration-time curve [AUC(0-infinity)] and peak concentrations of the drug “lignocaine” is increased by both “erythromycin” and “lignocaine”.

**Table 6:** Example sentences with PK DDI from literature

PMID	Study type	Sentence with DDI
10193676	in vivo	Both **erythromycin** and **itraconazole** increased the area under the **lignocaine** plasma concentration-time curve [AUC(0-infinity)] and **lignocaine** peak concentrations by 40-70 % (*P*<0.05).
10923859	in vitro	**Rifalazil**-25-deacetylation in microsomes was completely inhibited by **diisopropyl fluorophosphate**, **diethyl p-nitrophenyl phosphate** and **eserine**, but not by p-chloromercuribenzoate or 5,5′-dithio-bis(2-nitrobenzoic acid), indicating that the enzyme responsible for the rifalazil-25-deacetylation is a B-esterase.

The drug names involved in a PK DDI relation in each example are bolded

In vitro PK DDI dataset: 210 abstracts of in vitro PK DDI studies are included in the dataset. Different from in vivo studies, the conduct of in vitro DDI studies is used for determining whether a drug is a substrate, inhibitor, or inducer of metabolizing enzymes. By using in vitro technologies, it can qualitatively provide insight into the potential DDI based on the observation of enzyme kinetics parameters. Along with those PK data, a modeling or simulation approach is applied to describe the mechanism of drug interaction. An example sentence of in vitro PK DDI is displayed in Table 6, in which the metabolism of drug “Rifalazil” is inhibited by “diisopropyl fluorophosphates”, “diethyl p-nitrophenyl phosphate” and “eserine”, respectively.

All the drug pairs co-occurring in one sentence are considered as candidate DDI pairs. The interaction relations between drug pairs are labeled as “DDI” (positive) or “NDDI” (negative). Table 7 shows the statistics of the two datasets.

**Table 7:** Statistics of PK DDI datasets

	Dataset	Abstract	Sentence	Relation Pair	True Pair
in vivo	train	174	2114	2410	781
	test	44	546	889	207
in vitro	train	168	1894	4528	544
	test	42	475	1015	160

### Sentence representation

Sentences with candidate DDI pairs are represented at three linguistic levels, ranging from the dependency syntactic structure, shallow semantic relation structure and fine-grained semantic classes. For generalization, specific drug names in a candidate drug pair are replaced with “drug” in a preprocessing step. Take the sentence *S*_*1*_ as an example:

- *S*_*1*_: The inhibition of P-glycoprotein-mediated tubular secretion of Quinidine by Itraconazole.

The drug names “Quinidine” and “Itraconazole” are replaced with “drug1” and “drug2” before sentence representation.

#### Dependency graph

Dependency graph of a sentence is constructed on its dependency-based syntactic parse structure. It is a directed graph that includes two types of vertices: a word vertex contains its lemma and part-of-speech tags (POS), and a dependency vertex contains the dependency relation between words. In addition, both types of vertices contain their positions, which differentiate them from other vertices. Figure 3a illustrates the dependency graph of *S*_*1*_. Since the words connecting the candidate entities in a syntactic representation are particularly likely to carry information regarding their relationship [47], the labels of the vertexes on the shortest undirected paths connecting drug1 and drug2 are differentiated from the labels outside the paths using a special tag “IP”. Further, the edges are assigned weights; all edges on the shortest paths receive a weight of 0.9 and other edges receive a weight of 0.3 as in [32]. Thus, the shortest path is emphasized while also considering the other words outside the path as potentially relevant.

#### Shallow semantic graph

Shallow semantic graph uses predicate-argument structures (PASs) as shallow semantic representation of the sentence [48]. A predicate usually refers to a word indicating a relation or an attribute, and arguments refer to syntactic constituents with different semantic relations to the predicate [36]. For example, the preposition “by” in *S*_*1*_ is one predicate, “the inhibition of P-glycoprotein-mediated tubular secretion of drug1” is ARG1, representing the action being executed (denoted as by_arg1_), and “drug2” is ARG2, representing the executor of the inhibition (denoted as by_arg2_). Normalized PAS can be extracted from different surface textual forms by shallow semantic parsing [37].

The PAS employed in this study is defined by the Sign-based Construction Grammar [49]. The PAS graph is generated in the similar way as the dependency graph, except that the dependency vertex is replaced with a PAS vertex containing the relation between a predicate and its argument. If an argument is a phrase, an edge is connected from the predicate to the headword of the argument phrase. The PAS graph of *S*_*1*_ is illustrated in Fig. 3b. The shortest PAS path connecting drug1 and durg2 is “Inhibition of secretion of drug1 by drug2”; while the shortest dependency path is “drug1 by drug2” as shown in Fig. 3a. Dependency graph fails to include this critical information regarding DDI’s shortest path, when two drugs are connected by prepositional structures. In contrast, PAS graph can cover such information more comprehensively.

#### Semantic class annotation

In addition to dependency syntactic and shallow semantic relation structures, important terms involved in the PK DDI process are categorized into several semantic classes, such as “Drug”, “Enzyme”, “PK parameters”, “Change” etc. Table 8 displays the definitions and examples of each semantic class. Specifically, both the drugs and the metabolites of drugs are included in the “Drug” semantic class, which could involve a DDI relation. PK parameters are defined in the in vivo and in vitro PK ontologies by [24]. The “Mechanism” semantic class contains trigger words involved in PK DDI mechanisms. As an illustration, by replacing the specific terms in a sentence into the more generic semantic classes, *S*_*1*_ is converted to “The *Mechanism* of *Enzyme*-medicated tubular secretion of drug1 by drug2”. More details of those semantic classes can be found in [24].

**Table 8:** Semantic class description for literature of PK DDI

Semantic class	Definition	Example
Drug	Drugs, metabolites	quinidine
Enzyme	CYP450 enzymes	CYP1A2
PK parameter	PK Parameters	AUC
Number	Dose, sample size, values of PK parameters	40–70 %
Mechanism	Trigger words related to DDI mechanisms	stimulate
Change	Change of PK parameters	decrease
Degree	Severity of PK parameter change	strongly
Negation	Negative expression	negligible

Moreover, to differentiate among distinct mechanisms involved in PK DDI, and consequently reduce noisy features, the “Mechanism” class is further refined into three categories, as listed in Table 3: (1) The action of a drug on an enzyme; (2) The action of an enzyme on a drug; (3) The action converting a drug to its metabolite. Take sentence *S*_*2*_ as an example:

- *S*_*2*_: Drug1 inhibits the CYP2C19 -catalyzed 4-hydroxylation of drug2.

Here, “inhibits”, “catalyzed” and “4-hydroxylation”can be categorized into mechanisms of “Drug-enzyme”, “Enzyme-drug” and “Drug-metabolite”, respectively.

### All-path graph kernel

A graph kernel calculates the similarity between two input graphs by comparing the relations between common vertices. The weights of the relations are calculated using all possible paths between each pair of vertices. Our method follows the all-paths graph kernel proposed by Airola et al. [32]. The kernel represents the target pair using graph matrices based on two sub-graphs. The first sub-graph represents the structure of a sentence. Dependent on the type of structure representations of a sentence, two types of all-path graph kernels are employed in this study: (1) *Dependency graph kernel*, which is employed in the original all-path graph kernel, uses the dependency graph to represent sentence structure in the syntactic level; (2) *PAS graph kernel*, is a novel graph kernel defined in this study and uses the PAS graph to represent sentence structure at the shallow semantic level. Furthermore, semantic classes, representing the sentence content at a fine-grained semantic level, can be integrated into both dependency and PAS graph kernels by replacing the word vertices with semantic class vertices. As an illustration, Fig. 3c displays the dependency graph integrated with semantic classes of *S*_*1*_. The second sub-graph represents the word sequence in the sentence, and each of its word vertices contains its lemma, its relative position to the target pair and its POS; all edges receive a weight of 0.9 as in [32] (see Fig. 3d).

Assuming that *V* represents the set of vertices in the graph, calculation of the similarity between two graphs uses two types of matrices: edge adjacent matrix *A* and label matrix *L*. The graph is represented with the adjacent matrix *A* ∈ *R*^|*V*| × |*V*|^ whose rows and columns are indexed by the vertices, and [*A*]_*i*,*j*_ contains the weight of the edge connecting *v*_*i*_ ∈ *V* and *v*_*j*_ ∈ *V* if such an edge exists, and 0 otherwise. In addition, the labels are presented as a label allocation matrix *L* ∈ *R*^|*I*| × |*V*|^, so that *L*_*i*,*j*_ = 1 if the *j-th* vertex has the *i-th* label, and *L*_*i*,*j*_ = 0 otherwise. Using the Neumann Series, a graph matrix *G* is calculated as:

1 $$ G={L}^T{\displaystyle {\sum}_{n=1}^{\infty }{A}^nL={L}^T\left({\left(I-A\right)}^{-1}-I\right)L} $$

This matrix sums up the weights of all the paths between any pair of vertices, where each entry represents the strength of the relation between a pair of vertices. Given two instances of graph matrices *G*′ and *G*″, the graph kernel *K*(*G*^'^, *G*^' '^) is defined as follows:

2 $$ K\left({G}^{\hbox{'}},{G}^{\hbox{'}\hbox{'}}\right) = {\displaystyle {\sum}_{i=1}^{\left|L\right|}{\displaystyle {\sum}_{j=1}^{\left|L\right|}{G}_{ij}^{\hbox{'}}{G}_{ij\hbox{'}}^{\hbox{'}\hbox{'}}}} $$

### Experiments

#### Machine learning algorithm

Support vector machine (SVM) algorithms are the dominant ML methods (Segura-Bedmar et al., 2013) among the existing DDI systems. Our study used the sparse version of RLS, also known as the least squares SVM, to learn the DDI prediction model based on the all-path graph kernel [32].

#### Experimental setup

POS-tags and dependency trees of the datasets were generated using the Stanford parser [50]; PASs were generated by Enju [51], a deep parser based on a wide-coverage probabilistic HPSG grammar [52]. The semantic classes were annotated using pre-built lexicons and regular expressions [24]. Candidate drug pairs with two identical drugs were removed from the training and test datasets.

We used the standard evaluation measures (Precision, Recall and F-measure) proposed by the DDI extraction challenge [19] and employed previously on the same PK DDI dataset used in our study by [24] to evaluate the performance of our system.

The package of the all-path graph kernel algorithm provided in [32] was employed in our experiments. Built on the lease squares SVM, this package provides configuration options for some SVM parameters, as well as graph kernel related parameters. In addition, to find the optimal threshold for prediction in the generated model, a leave-one-document-out cross validation function is provided. Thus, cross-validations were first conducted on the training datasets. Relation extraction models were then built on the training datasets, using the optimal thresholds for prediction. The performance on test datasets was evaluated using those models and reported. Currently, data vectors were created without normalization, which dropped the performance in our pilot study; 500 basis vectors were used for model building. For graph kernels, all edges on the shortest paths received a weight of 0.9 and other edges received a weight of 0.3. For the word sequence based kernel, all edges received a weight of 0.9.

Experiments and systematic analysis were conducted as follows:

1. Graph kernels of syntactic and shallow semantic representations: dependency graph kernel (DEP) and shallow semantic graph kernel, i.e., PAS graph kernel were employed in this study, as described in the METHODS section. The dependency graph kernel, which was used in the original all-path graph kernel [32], served as the baseline in this study. The difference in performance between the syntactic and shallow semantic graphs was examined.
2. The combination of graph kernels with semantic class: To evaluate the effect of semantic class (SC), it was incorporated into each graph kernel, as described in the METHODS section.
3. Different granularities of the “Mechanism” semantic class: In order to check whether differentiating among distinct mechanisms would influence the performance, the refined semantic classes of “Mechanism” as defined in Table 3 were incorporated into graph kernels, along with other semantic classes (ReSC).

For systematic analysis, pairwise t-tests were conducted between the results of all proposed methods and the baseline method (DEP). Besides, pairwise t-tests were also conducted between the results of PAS_SC/PAS_ReSC and PAS, to examine the improvement of incorporating semantic class information with PAS. The statistical significance (p-value < 0.05) of the proposed methods was evaluated both on the in vivo and in vitro datasets. Furthermore, using scores output by the prediction models as thresholds, ROC curves of the implemented methods were also constructed for the in vivo and in vitro datasets, respectively.
